# ‘… a metal conducts and a non-metal doesn't’

**DOI:** 10.1098/rsta.2009.0282

**Published:** 2010-03-13

**Authors:** P. P. Edwards, M. T. J. Lodge, F. Hensel, R. Redmer

**Affiliations:** 1 Department of Chemistry, Inorganic Chemistry Laboratory, University of Oxford, South Parks Road, Oxford OX1 3QR, UK; 2 Fachbereich Chemie, Philipps-Universität Marburg, Hans-Meerwein-Strasse, 35032 Marburg, Germany; 3 Institut für Physik, Universität Rostock, 18051 Rostock, Germany

**Keywords:** metals, metal to non-metal transitions, doped semiconductors, periodic table of chemical elements

## Abstract

In a letter to one of the authors, Sir Nevill Mott, then in his tenth decade, highlighted the fact that the statement ‘… a metal conducts, and a non-metal doesn’t’ can be true only at the absolute zero of temperature, *T*=0 K. But, of course, experimental studies of metals, non-metals and, indeed, the electronic and thermodynamic transition between these canonical states of matter must always occur above *T*=0 K, and, in many important cases, for temperatures far above the absolute zero. Here, we review the issues—theoretical and experimental—attendant on studies of the metal to non-metal transition in doped semiconductors at temperatures close to absolute zero (*T*=0.03 K) and fluid chemical elements at temperatures far above absolute zero (*T*>1000 K).

We attempt to illustrate Mott’s insights for delving into such complex phenomena and experimental systems, finding intuitively the dominant features of the science, and developing a coherent picture of the different competing electronic processes. A particular emphasis is placed on the idea of a ‘Mott metal to non-metal transition’ in the nominally metallic chemical elements rubidium, caesium and mercury, and the converse metallization transition in the nominally non-metal elements hydrogen and oxygen. We also review major innovations by D. A. Goldhammer (Goldhammer 1913 *Dispersion und absorption des lichtes*) and K. F. Herzfeld (Herzfeld 1927 *Phys. Rev.* **29**, 701–705. (doi:10.1103/PhysRev.29.701)) in a pre-quantum theory description of the metal–non-metal transition, which emphasize the pivotal role of atomic properties in dictating the metallic or non-metallic status of the chemical elements of the periodic table under ambient and extreme conditions; a link with Pauling’s ‘metallic orbital’ is also established here.

## 1. Prologue: Sir Nevill Mott

**Figure 1.**
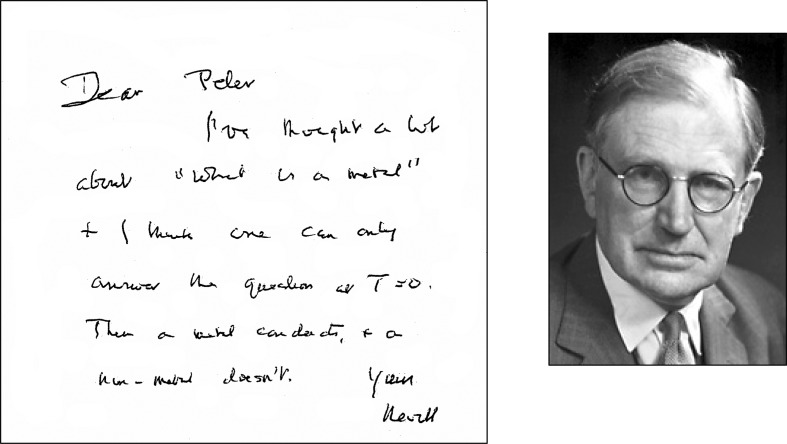
Sir Nevill Mott: ‘… a metal conducts and a non-metal doesn’t’. A letter from Sir Nevill Mott to P. P. Edwards 9 May 1996: Nevill answers the question, ‘What is a metal?’. (Reproduced with permission from Edwards 1998.)

Over a period of more than half a century, Prof. Sir Nevill Mott pioneered the development of key concepts, models and theories for discussing the fundamental problem of metals versus non-metals (insulators and semiconductors).^1^  These issues occupied the thoughts of Sir Nevill well into his nineties. In figure 1, we reproduce part of a letter written to one of us (Prof. Peter P. Edwards) on Thursday 9 May 1996, in which he notes:

Dear Peter, I’ve thought a lot about ‘What is a metal?’ and I think one can only answer the question at *T*=0 [the absolute zero of temperature]. There a metal conducts, and a non-metal doesn’t.

(Edwards 1998)

Of the many pieces of work showing Mott’s most profound insights (Edwards & Rao 1985, 1995; Davis 1998, 2007; Redmer *et al.* 2010), the metal–non-metal transition, generally called the Mott transition, is in our view a most influential contribution. Mott discussed this complex phenomenon not only in terms of a coherent theory, but also provided key operational experimental criteria for understanding the location of the Mott transition and its signature electronic features. Almost certainly, the very first attempts to explain the occurrence of metals and non-metals—in fact, predating the efforts of Mott—were made as remarkable contributions by Goldhammer (1913) and Herzfeld (1927). This old and, until recently, half-forgotten pre-quantum theory allows one to extract and use deep insights into the fundamental issues of atomic properties, and elemental densities which make an element or substance metallic or non-metallic. Here again, as we hope to illustrate, this approach is invaluable for the experimental study of complex phenomena in complex systems!

## 2. The electrical properties of matter

It has been known since the earliest studies of electrical currents in substances that metals conduct electricity well and most other materials do not. Just how remarkable are metals as ‘… magnificent conductors of electricity’ (Cottrell 1991) can be gauged from the enormous range of electrical conductivities that exist even at room temperature between the most conductive of materials (copper and silver) and some of the most resistive (Ehrenreich 1967) (e.g. glass and diamond).

It has been suggested that this variation in electrical conductivities, at least some 28 orders of magnitude, represents the widest range of any observable physical property and this does not include the phenomenon of superconductivity (Kittel 1976). This difference between metals and non-metals becomes even more pronounced as one goes to lower and lower temperatures. For non-metals, the electrical conductivity rapidly decreases as temperature decreases; ultimately, the substance becomes completely non-conducting at the absolute zero of temperature, i.e. the resistivity levels to infinity. For metals and also for other metallic substances, such as certain oxides and heavily doped semiconductors, it is the opposite; here, the electrical conductivity increases with decreasing temperature and tends to a finite value at the limit of *T*=0 K. In this work, following Mott, we therefore use the term ‘metal’ to describe materials and substances in which the conductivity tends to a finite value for *T*=0 K, and ‘non-metal’ to describe those for which the conductivity tends to zero at the limit of *T*=0 K. A central focus of this paper is also to understand the situation for temperatures far above absolute zero, and any ‘distinction’ one might hope to draw between metals and non-metals for *T*≫0 K, as in the case for expanding metals at high temperature (Hensel & Warren 2000) or, perhaps even more exotic, for the warm dense matter in giant planets at elemental densities exceeding normal densities and temperatures of several electron volts.

Following the discovery of the electron by J. J. Thomson in 1897 and the realization that it was a universal constituent of all matter, the phenomenon of metallic conductivity was ascribed to the electron by P. Drude and A. H. Lorentz at the beginning of the last century (Springford 1997). A metal was pictured as a framework of ions through which itinerant electrons made their way, under the influence of an electric field, much as they do in gas discharges. The actual resistivity of a metal was viewed as being caused by collisions between these itinerant electrons and parent ions.

Drude assumed that, in a metal, these free electrons form a kind of ‘electron gas’. It is worth remembering that, in 1900, the electron was still an entirely new concept and the picture of conductivity being ascribed to electronic motion was a new—and, of course, correct—idea (Edwards 1968; Mott 1980*a*). Bernal (1929) further broadened the discussion by noting that the widest definition of metals *and* the metallic state was of a substance transmitting electricity by electron transfer. The application of quantum mechanics to the Drude model, by Sommerfeld and then Bloch, provided the first quantized free-electron picture which, even a century later, still remains the cornerstone of our thinking about how we describe a metal (Kittel 1976; Hoddeson & Baym 1980). But what about insulators? Here, presumably, electrons were not free but, rather, stuck!

In this regard, Sir Nevill Mott (1984) recounted his experiences of attending a lecture course on ‘Electron theory of metals’ in 1926, in Cambridge:

I remember asking the lecturer why electrons are free in some materials and not in others. Of course he did not know. We just had to think that in insulators the electrons were ‘stuck’.

(Mott 1984)

The problem of why some elements, substances or materials are metallic and others are not lingered through most of the last century. Bardeen (1940) noted that ‘There is no satisfactory explanation on any classical basis’. Reflecting the sentiments of Bardeen (1940), it is indeed generally assumed that, until the advent of quantum mechanics, no clear understanding was possible as to why the electrons were stuck in non-metals but were free in metals. This viewpoint is not correct; one should note quite remarkable earlier advances in the pre-quantum mechanical models of the difference between metals and non-metals by Goldhammer (1913) and Herzfeld (1927), based on classical mechanics. For our present purposes, though, we will return later (§4) to these appealing pre-quantum mechanical models based on the role of atomic properties and elemental density on the metallic and non-metallic states of matter.

**Figure 2.**
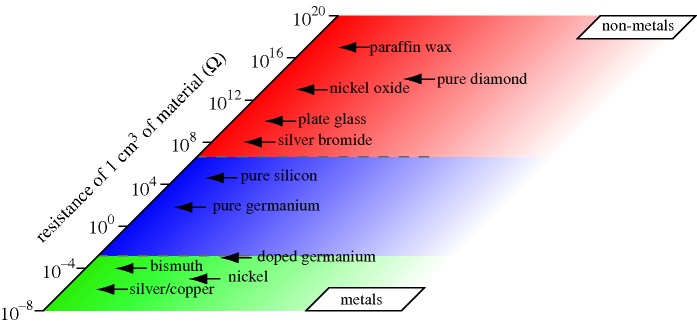
The room-temperature electrical resistivity of materials and substances is one of the most widely varying of all physical properties, encompassing 28 orders of magnitude difference between metals and non-metals. At the absolute zero of temperature, non-metals do not conduct, while metals do (excluding here the phenomenon of superconductivity). (Adapted from Ehrenreich 1967.)

Here one should also note the critically important ideas of Pauling as to the *nature of the chemical bond in metals* (Pauling 1938, 1949, 1960, 1981). Pauling recognized that the most striking characteristic features of the chemical bond that holds atoms together in a metal is indeed the high mobility of the bonding electrons, which also gives rise to the high electrical (figure 2) and thermal conductivity of metals. Pauling’s ‘metallic orbital’ defined an extra orbital (over and above the local bonding requirements), which permitted the unsynchronized resonance of electron-pair *bonds*, from one interatomic position to another, leading to great stabilization of the metal by ‘resonance structures’ and to the characteristic properties of a metal.

 Feynman *et al.* (1970) also alluded to these fundamental problems in their celebrated *Lectures on Physics*, noting in the introductory remarks ‘Some [materials] are electrical ‘conductors’–because their electrons are free to move about: others are ‘insulators’ (non-metals)–because their electrons are held tightly to atoms. We shall consider later how some of these properties come about, but that is a very complicated matter’. Interestingly, it is not instantly obvious that Feynman and colleagues (1970) did revisit this ‘… very complicated matter’, in that famous text! We now take up this challenge and ask, ‘Why is it that some substances are metals (conductors), while others are non-metals (non-conductors)?’

## 3. Energy bands for metals, non-metals and semiconductors

One of the earliest recognized successes of the quantum theory of solids was the explanation by Wilson (1931*a*,*b*) of the reason for the sharp distinction in nature between metals and non-metals. In crystalline materials, the quantum states of electrons lie in bands; non-metals are elemental substances or materials in which all the electronic energy bands are either full or empty; metals possess bands which are only partly full.

**Figure 3.**
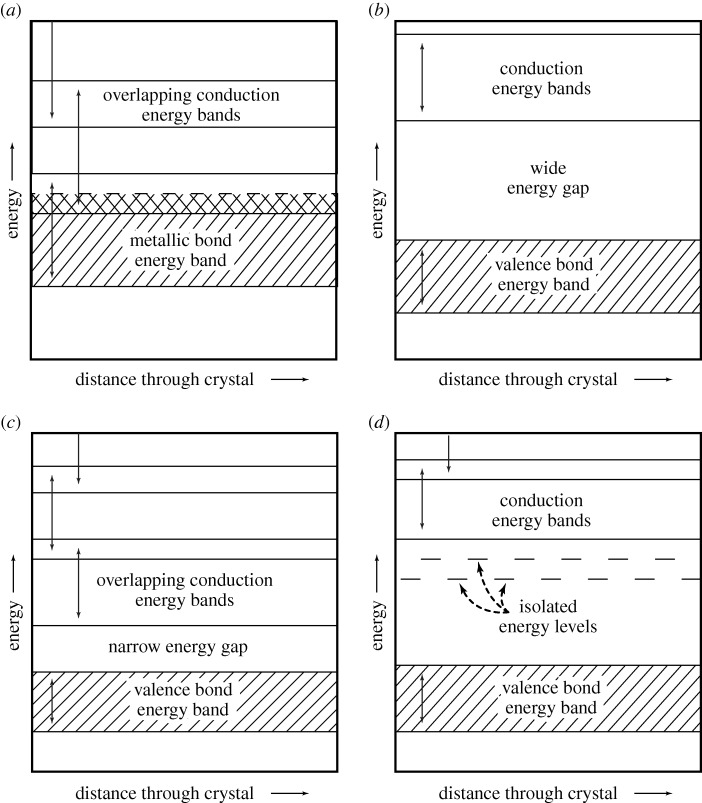
Energy band diagrams for (*a*) a metal, (*b*) an insulator (non-metal), (*c*) an intrinsic semiconductor and (*d*) an impurity semiconductor. (Adapted from Shockley 1950.)

Wilson (1931*a*,*b*) demonstrated that from these characteristic differences between the energy bands of metals and non-metals (and semiconductors) their electrical properties can be understood. The energy-level schemes for the cases of metals, insulators (non-metal) and intrinsic semiconductors are shown in figure 3.

When all of the quantum states of electrons in an electronic band are occupied, the band electrons cannot participate in conduction. Interestingly, in this model, this is not because the electrons are trapped or stuck, but because exactly as many free electrons are moving from left to right as from right to left upon the application of an electric field.

In the case of a metal such as copper or silver, the energy bands overlap in such a way that they are occupied by the available electrons, but some bands are left only partially occupied. On the other hand, in pure diamond, for example, one set of energy bands is occupied by valence electrons, and the next higher set (the conduction band) is left entirely empty. For diamond, the energy gap between them is so great that electrons are not thermally excited, and diamond, therefore, has either completely filled or completely empty energy bands and is a non-metal (insulator) when in thermal equilibrium at room temperature. The magnitude of a substance’s electrical conductivity/resistivity can then be understood, in part, by the magnitude of its characteristic energy gap, and potential thermal excitation of carriers to the conduction band, any remaining positive ‘holes’ in the valence band (figure 3) also contributing to the conductivity.

The diamond form of tin, sometimes called grey tin (usually stable below room temperature), is probably either a metallic conductor with overlapping energy bands or at least an intrinsic semiconductor of very high conductivity. An interesting graduation (Shockley 1950) in electronic energy gaps for tetravalent elements of group 14 is

**Table:** 

carbon (diamond)	silicon	germanium	tin (grey)	tin (metallic)	lead
6–7 eV	1.1 eV	0.72 eV	0.1 eV	(overlapping bands)	

It was perhaps typical of the almost instantaneous acceptance of the (then) new quantum mechanics that few doubted this intriguing model of the fundamental differences between metals and non-metals. Moreover, a basic approximation in the model for both metals and non-metals was that each electron moved independently of all other electrons; that is, no account was taken of the electron–electron (Coulomb) repulsive interactions. Bloch (1980) himself admitted to an uneasy feeling that this model of independent electrons, i.e. a model in which electron–electron interactions are not considered, might represent a rather poor approximation, even in the case of highly conducting free electrons in metals.

Although widely accepted at the time, this criterion has since been proved to be incomplete. In particular, it was recognized that there are classes of materials which, if described according to Wilson’s (1931*a*,*b*) scheme, should be metallic conductors but are in fact non-metals.

Thus, de Boer & Verwey (1937), in one of the earliest studies of the electrical properties of transition metal oxides, pointed out that nickel oxide, for example, is a green semiconductor, which according to the Wilson model should be a metallic conductor owing to the fact that the eight d electrons of the Ni^2+^ ion would only partly fill any 3d electronic band. In the discussions following de Boer & Verwey’s (1937) paper, Mott & Peierls (1937)^2^ built upon some of those authors’ insightful remarks to suggest that strong Coulomb repulsions between d electrons on adjacent Ni^2+^ ions could lead to localization of electrons at individual sites, rather than delocalization throughout the entire solid, as would be expected with the Wilson scheme.

 Mott (1937) recognized this example as symptomatic of the critical role that electron–electron interactions or correlations must play in any description of electrons in solids (and liquids). We now understand that the effects of electron correlations in certain systems can cause them to be insulating when they should be metallic according to band theory. In a determined campaign starting in 1949, Mott urged that this problem be recognized for what it was—a fundamental challenge to solid-state theory (Mott 1949, 1956, 1958, 1961, 1982; Mott & Stevens 1957). Mott thereby laid the foundations of a deep physical understanding of the difference between metals and non-metals and with that the realization of the potential transition between these two canonical states of matter, the metal–non-metal transition (Mott 1990). His contributions were the launching pad for a fascinating, and still developing, field of science.

## 4. Interacting or correlated electrons and the Mott transition

In attempting to get to a tractable and realistic physical model—a hallmark of Mott’s approach—for the role of electron correlation and other concepts for discussing the metallic and non-metallic states of matter, Mott (1956, 1958, 1982, 1990) uncovered a rich, fascinating and most important subject, that of the metal–non-metal transition. Remarkably, there are now numerous experimental examples where highly conducting metals and metallic systems can transform into stubbornly resistive non-metals, and vice versa. If the issue of metals versus non-metals is indeed a ‘complicated subject’ (according to Feynman *et al.* (1970)), what then of the situation where each of these phases can transform one to the other… the metal–non-metal transition?

Such transitions can be induced by continuous changes in thermodynamic control parameters such as temperature, composition and pressure (Edwards & Rao 1985, 1995; Edwards *et al.* 1998; Imada *et al*. 1998; Redmer *et al.* 2010). Indeed, it is common to find the electrical conductivity in many systems and substances changing by factors of between 10^3^ and 10^10^ over quite small ranges of these thermodynamic parameters. To appreciate the insight, intuition and tractable formalism developed by Mott, we briefly review his approach to the vexing problem of interacting or correlated electrons and the so-called Mott metal–non-metal transition.

### (a) The case at T=0 K

In order to consider the precise conditions under which the Wilson approach might break down, Mott, in a series of papers beginning in 1949, considered an array of hydrogen or other monovalent atoms (e.g. the alkali elements) separated by an interatomic distance, *d*. He then discussed in detail how one would attempt to describe the electrons in such an array, and how any description must depend critically on the value of the interatomic distance. Consider his intuitive approach in the following way. If *d* is sufficiently large, and the resulting orbital overlap between the valence electrons on neighbouring hydrogen or alkali atoms is negligibly small, each electron is surely best described by an *atomic* wave function. The assembly of any such atoms is obviously (intuitively) non-conducting at *T*=0 K: electrons can of course change places (electron exchange), but no net electric current flow would surely be possible. To introduce electron transport throughout this assembly, ‘polar’ ionic states would need to be introduced into the ground state wave function in such a way so as to allow the passage of electric current. In other words, electron conduction within an assembly of such neutral, non-interacting atoms would require the ionization of an electron from one of the atoms and its subsequent transfer to another (neutral) atom in the assembly.

This aspect is now formally embodied within the so-called Mott–Hubbard correlation energy (*U*), which is the magnitude of the energy difference between the ionization energy (*I*) and the electron affinity (EA) of the isolated atom. Mott noted that the energy *U* (=*I* – EA) then represents the extra energy cost of putting two electrons (instantaneously) on any one atomic site, and an activation energy of this order is necessary for conduction in the limit of large *d*. Hubbard later introduced a detailed model in which the Coulomb interactions between electrons are explicitly included only when they are on the same atom (Hubbard 1963, 1964). For large values of *d*, this interaction splits the electronic band, so that an electron’s energies lie in a full band and an empty one (the so-called upper and lower Hubbard bands).

Now, according to the Wilson model, such an assembly of hydrogen-like atoms (or indeed more generally atoms or ions in which there is an incomplete electronic shell) ought to be a metallic conductor *for all values of d*, even though the electronic band would obviously narrow and the effective mass increase with *d*. Mott (1961) noted, incisively, ‘…this is against common experience and, one might say, common sense’.

If we look at the corresponding situation for small separations of the atoms, particularly those separations for which *d* is comparable to, or less than, the known value for the metallic element, one surely has metallic character at *T*=0 K and a free-electron wave function. Mott (1949, 1956, 1958, 1961, 1982) went further and proposed a transition from metallic- to atomic-like wave functions as *d* is continuously increased. He presented arguments to show that the change from a conducting (metallic) to a non-conducting (atomic) state must be sharp: interestingly—and controversially—he proposed that, depending on the value of *d*, either all of the electrons are free to move, or none are (Mott & Stevens 1957).

Mott’s prediction was that, as the distance between atoms in this hypothetical assembly is continuously increased, there will, at the absolute zero of temperature, be a sharp transition from a metallic state having a finite (or infinite) conductivity at *T*=0 K to a non-metallic or insulating state in which there is no conduction at the absolute zero of temperature; hence, his potent intuitive statement noted at the very beginning of this article (figure 1). (Indeed, much earlier, Wigner (1938) had also suggested that an electron gas of low concentration might ‘crystallize’ the electrons becoming localized in space.)

 Mott (1961) further proposed that, at a certain critical density (*n*_c_) of atoms, which of course corresponds to a critical distance (*d*_c_) between the atoms, a first-order, discontinuous transition from a metal to a non-metal would occur at *T*=0 K. The question was at what value of *d*_c_ would such a metal–non-metal transition occur? His assumption was that this would occur when the (screened) potential around each positive charge
4.1
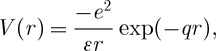

with the screening constant *q* calculated from the so-called Thomas–Fermi model, was just sufficient to trap a free electron from the metallic gas. The quantity *q* increases with *n*, and if *q* is large enough the potential hole becomes too small to allow a bound state to form.

With varying atom density *n*_c_, and thus *q*, Mott (1961, 1990) proposed that there would be a discontinuous transition from an electronic state with all the valence electrons trapped on individual atoms to one where all are free and this metal to non-metal transition would occur when (Edwards & Sienko 1978)
4.2
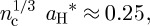

where *a*_H_^*^ is the hydrogenic radius of the isolated (low density) atomic state (in this instance).

From equation (4.2), we see that, in the ultimate transition to the metallic state, free electrons—and attendant metallization—do not form until the mean interatomic distance between atoms is below about four multiples of the hydrogen radius of any one of them.

This conclusion, namely that such a collection of hydrogen-like atoms would inevitably undergo a metal–non-metal transition, was perhaps not surprising: what was remarkable, however, was Mott’s (1958, 1961) conjecture that, at a critical distance *d*_c_ (equivalent to a density *n*_c_), *all* valence electrons would become localized at once (Mott & Stevens 1957). In the corresponding non-metal–metal transition, all valence electrons and not just a few of them would therefore be set free at *d*_c_ (*n*_c_).

**Figure 4.**
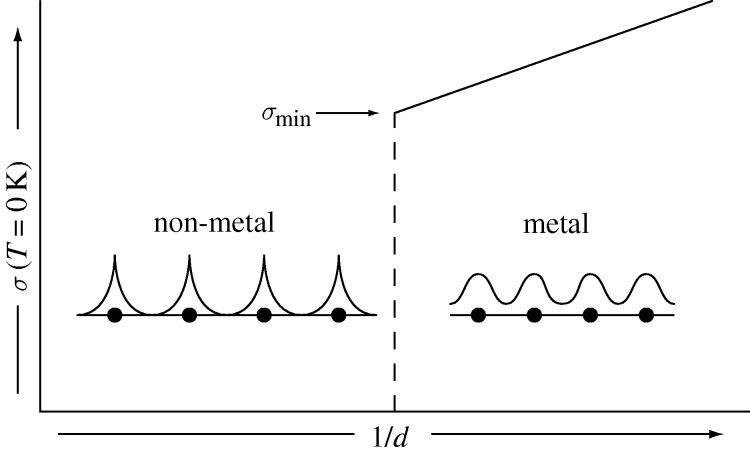
The metal–non-metal transition at *T*=0 K, whereby free (conduction) electrons become localized at individual sites. The system transforms *discontinuously* from a metal to a non-metal as the mean distance between centres continuously increases.

This kind of first-order, discontinuous electronic phase transition from metal to non-metal (or vice versa) at *T*=0 K has been known as ‘the Mott transition’. The ramifications of such a theoretical Gedanken experiment for a hypothetical assembly of hydrogen-like centres in which the interatomic distance is continuously varied are shown in figure 4.

 Mott (1972) subsequently argued that, at the very transition from a metal to a non-metal, there would exist a so-called ‘minimum metallic conductivity’ 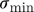, which would signify the minimum value of the DC electrical conductivity at the *T*=0 K transition. Importantly, this is the minimum value of the electrical conductivity for which the substance can still justifiably be viewed as a metal, prior to the complete localization of the gas of itinerant, free electrons as the system moves into the non-metallic, insulating state.

This intriguing concept has its origins in the vastly important 1958 paper by P. W. Anderson (Anderson 1958, 1978) entitled ‘On the absence of diffusion in certain random lattices’. As Mott noted, this paper had a profound effect on our understanding of the behaviour of electrons in non-crystalline, or disordered, media. Anderson showed that sufficient disorder in a material or substance can produce localization of the electronic states within an energy band, whereby the familiar extended states of Bloch become localized in space. Mott (1972) outlined what we expect at such an ‘Anderson metal to non-metal transition’; as we increase the strength of the electron scattering by disorder, we reach a limit at which Anderson localization of the electronic energy states sets in. The conductivity just before this transition occurs is Mott’s minimum metallic conductivity.

Figure 4 then reflects the idea of a discontinuous drop in conductivity from a value of 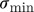 to zero at the metal–non-metal transition occurring at the absolute zero of temperature. From such arguments, and following , one indeed expects a sharp distinction in nature between metals and non-metals for temperatures approaching the absolute zero. Thus, a metal would exhibit a finite, or infinite, value of the DC electrical conductivity while a non-metal would have zero conductivity at *T*=0 K. Such a *discontinuous* metal–non-metal transition is, and still remains, a remarkable and tantalizing theoretical prediction, and has long been sought (Edwards *et al.* 1998, 2000) by experimentalists and theoreticians alike!

This fundamental distinction between the metallic and non-metallic states of material is vividly illustrated by ‘zero-temperature’ conductivity measurements by Rosenbaum *et al.* (1980) on large single-crystal ingot samples of the semiconductor silicon doped with elemental phosphorus (Si : P), down to temperatures of just 0.030 K, and the results further extrapolated down to *T*=0 K! The rapid drop in the resistance of most semiconductors that occurs when the concentration of ‘impurity’ or ‘dopant’ atoms exceeds a certain value was well known and Mott described this as one important example of the transition to the metallic state.

**Figure 5.**
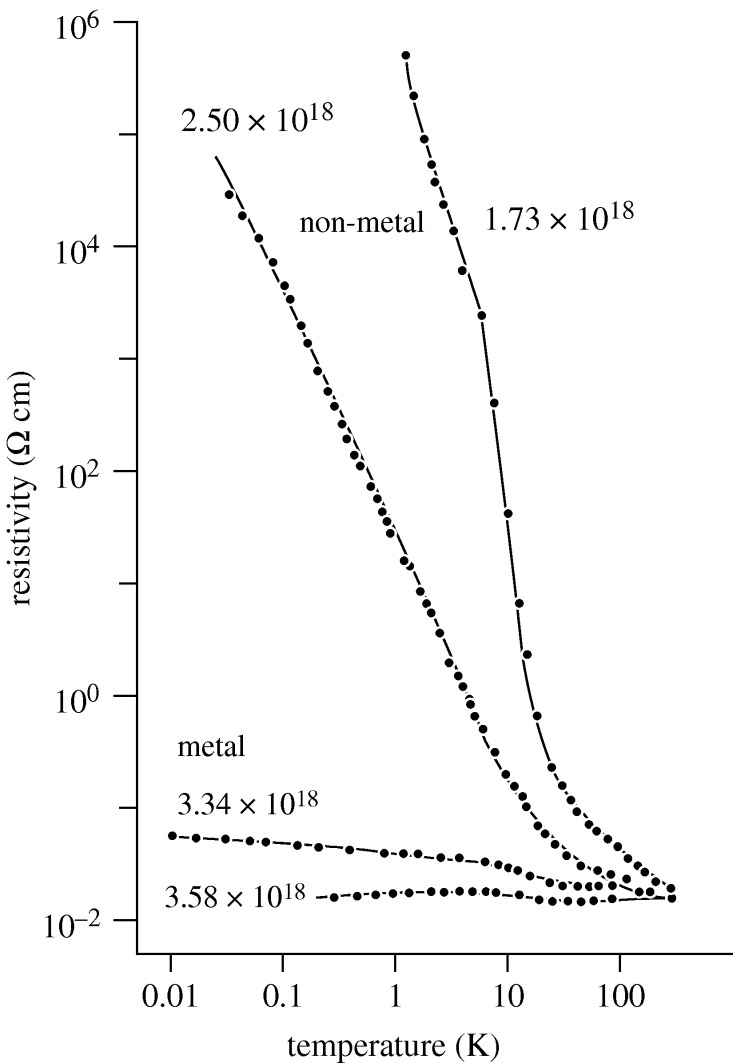
The measured electrical conductivities of a range of bulk crystals of silicon doped with phosphorus (Si : P). The electron (or donor atom) density supplied by the phosphorus donor atoms is indicated on each curve in units of electrons per cubic centimetre.

**Figure 6.**
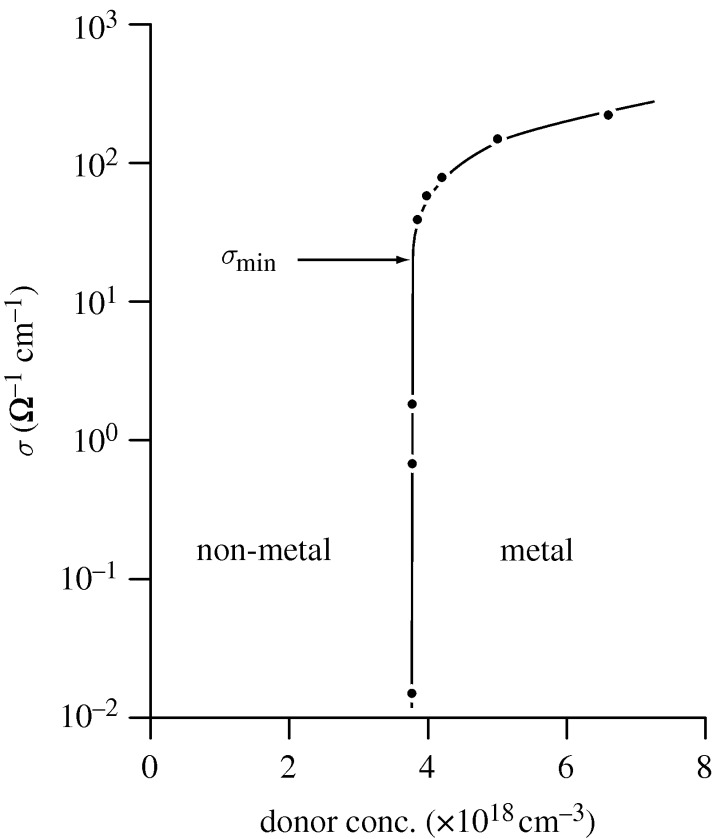
A semi-logarithmic plot of the zero temperature conductivity, *σ* (0), versus the donor atom content for Si : P. The transition from non-metal to metal is extremely sharp, but probably continuous. Mott’s minimum metallic conductivity is also indicated for this particular example. The critical density for the metal–non-metal transition is 3–4×10^18^ cm^−3^ (Rosenbaum *et al.* 1980).

In figures 5 and 6, it can be seen that the non-metal to metal transition in the semiconductor silicon doped with phosphorous occurs at an increasing dopant density (*n*_c_) of some 3×10^18^ electrons cm^−3^. For a sample with *n*<*n*_c_, the material is clearly a non-metal or insulator for *T*→0 K with a rising electrical resistivity as the temperature decreases. For *n*>*n*_c_, the material is a metal (having a finite conductivity for *T*→0 K). The metal–non-metal transition is undoubtedly sharp, but possibly continuous, as we see several samples having intermediate values of the conductivity between zero and 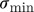 (figure 6). Mott’s estimate for 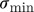 is also indicated. It is remarkable that these ‘fingerprint’ parameters for the location of the metal–non-metal transition, namely *n*_*c*_ and 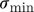, appear to be excellent indicators for the experimental situation close to *T*=0 K. However, we note here that even the apparently ‘simple’ temperature extrapolations from 0.03 to 0 K are still highly controversial!

The obvious question, and challenge, then arises as to how to attempt to understand the numerous experimental situations at considerably higher temperatures—sometimes far above *T*=0 K—where it is possible to transform a non-metallic material, having a low conductivity, to a highly conducting, metallic counterpart, through changes in composition, pressure, temperature and other parameters.

### (b) The case at T≫0 K (1000 K and above)

But how are we able to experimentally test Mott’s hypothesis for metallic and non-metallic substances at temperatures far above *T*=0 K? One important class of systems centres on the nominally metallic elements—rubidium, caesium and mercury—which under supercritical conditions (approx. 2000 K and 0.01 GPa) can be continuously probed via expansion from metallic densities to very low values characteristic of the vapour phase of the elements (Hensel 1990, 1998; Hensel & Warren 2000).

Transitions from non-metallic to metallic fluids have also recently been observed in the nominally non-metallic chemical elements hydrogen, oxygen and nitrogen when compressed to high pressures (100 GPa, equivalent to 1 Mbar) and temperatures in excess of several 1000 K (Nellis *et al.* 2003; Nellis 2004). Thus, under these extreme conditions, these various chemical elements should behave as metals at sufficiently high densities, and as non-metals at sufficiently low densities.

Remarkably, this possibility was identified at the dawn of the last century by Strutt (1902), who noted that ‘Mercury vapour is an insulator, while liquid mercury is a conductor. Since the liquid and saturated vapour are indistinguishable above the critical temperature, one or both of these must undergo a remarkable change of electrical properties as that temperature is approached.’

We review how the transition from metallic to non-metallic conduction can be observed in a highly expanded metallic fluid or a highly compressed non-metallic vapour; for this, we look in detail at how electronic properties of five chemical elements, hydrogen, oxygen, rubidium, caesium and mercury, vary with elemental density.

For three of these elements, hydrogen, rubidium and caesium, the link with Mott’s ‘Gedanken experiment’ is close and tantalizing in that above their respective critical points these elements undergo density-induced metal–non-metal transitions (Hensel & Edwards 1996). However, of course, these are extremely high temperature systems—far, one must say, from the *T*=0 K visualization so beloved by Mott! (And, of course, far from the extremely low temperature studies of Si : P.)

Nevertheless, these high-temperature systems represent excellent examples for investigating the metal–non-metal transition in chemically quite dissimilar elements, for the following reasons (Inui *et al.* 2007; Matsuda *et al.* 2007).

First, three of these elements, hydrogen, rubidium and caesium, are of course contenders for Mott’s prototypical array of hydrogen-like atoms for investigating the transition to/from the metallic state (Mott 1961). Second, in the supercritical state of these elements, the density can be continuously varied at will, allowing one to probe the density dependence of metallic versus non-metallic properties. Third, the ‘background’ positive ions in such fluids can undergo facile readjustment in their positions when compared with the corresponding situation in (low temperature) solid-state systems. This is leading to new insights (Inui *et al.* 2007; Ruland & Hensel in press) into the coupling of electrons and ions under varying conditions of density. Last, but certainly not least, rationalizing the respective metal–non-metal transition densities of these five quite dissimilar chemical elements under conditions of continuously changing density leads to a (welcome) degree of ‘universality’ in understanding how individual atomic properties make an element metallic or non-metallic under ambient conditions on this planet, or indeed on other planets.

There is now a wealth of experimental evidence which shows that most elemental metals that can exist in the fluid state under normal (i.e. room pressure) conditions, such as mercury, rubidium and caesium, become non-metallic when they are expanded to low densities. These studies probe the supercritical states of the various elements where the density can be continuously varied, allowing one to investigate the density at which each ‘metallic’ element actually becomes a metal—or, indeed, the situation when nominally non-metallic elements such as hydrogen and oxygen (designated as such from our room temperature, room pressure experience) transform to the metallic state. As well as the tremendous technical difficulties associated with experiments on these high-temperature systems, one is also forced to confront the basic conceptual problem. Namely, in systems at temperatures far above absolute zero, how can we distinguish between a metal and a non-metal? And, are the different density-dependent behaviours understandable in ‘simple’ models for the metal–non-metal transition, especially in light of the high temperatures *and* of course recognizing the fact that these are disordered liquids and vapours?

**Figure 7.**
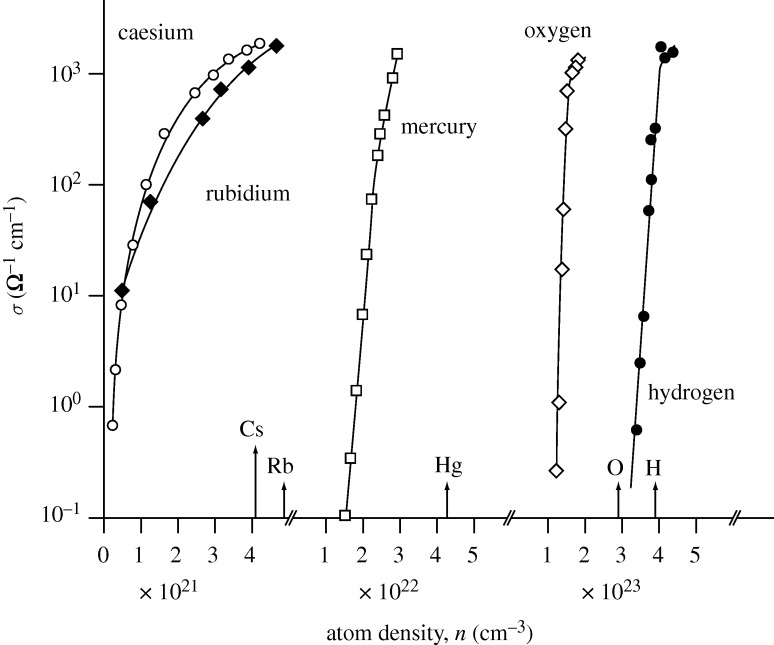
The electrical conductivity of fluid caesium, rubidium, mercury, oxygen and hydrogen versus atom density, *n*. The tag lines on the atom density axis denote the predicted metallization densities for each element, based on the Goldhammer–Herzfeld model (see text). Note that the experimented atom densities range from 10^21^ cm^−3^ for rubidium and caesium to over 10^23^ cm^−3^ for oxygen and hydrogen. (Adapted from Edwards & Hensel 2002.)

In figure 7, we highlight the evolution of the density-induced non-metal to metal transition for the high-temperature fluids of the chemical elements hydrogen, oxygen, rubidium, caesium and mercury. The conductivity data as a function of atom density, *n*, clearly illustrate the decisive role that the density of a chemical element plays in dictating whether that element exists as a metal or a non-metal. Note the very large changes in conductivity for elements conventionally designated as ‘metals’ (rubidium, caesium and mercury) and non-metals (hydrogen and oxygen). Indeed, one can now readily see that density is clearly one of *the* dominant thermodynamic variables governing the metallic versus the non-metallic nature of chemical elements of the periodic table.

To gain further insights into the electronic processes in operation, we outline a direct link between Mott’s view of metallization, Pauling’s concept of ‘the metallic orbital’ and an approach from even earlier in the last century, namely the work of Goldhammer (1913) and Herzfeld (1927) noted earlier (Logan & Edwards 1985).

Almost certainly, the very first attempts to explain the occurrence of metallic versus non-metallic behaviour is a chemical element—and of course with that the first-ever discussion of the metal–non-metal transition, which can be traced back to the remarkable work of Goldhammer (1913) and Herzfeld (1927). These classical approaches—namely pre-quantum mechanical approaches—were based on the concept of a density-induced dielectric catastrophe, whereby the dielectric constant of a substance diverges at the critical metallization density (the transition to the metallic state), causing the release (or wholesale freeing) of the bound valence electrons. This rationalization, in terms of atomic properties which in a sense confers metallic versus non-metallic status for a chemical element, leads to what is known as the Goldhammer–Herzfeld criterion for metallization.

This link can be viewed in terms of the Clausius–Mossotti relationship, (*n*^2^−1)/(*n*^2^+2)=*R*/*V* , where *n* is the index of refraction (the high-frequency dielectric constant), *R* is the molar refractivity (or sometimes called the mole refraction), given by ((4/3)*πNα*), where *N* is the Avogadro number, *α* is the atomic electronic polarizability and *V* is the molar volume. Herzfeld (1927) argued that if we start with an isolated, polarizable atom in the gas phase and transform it to the condensed liquid or solid phase, thereby continuously increasing the elemental density such that the ratio (*R*/*V*) increases, then at a critical condition (*R*/*V*)=1, one has the equality (*n*^2^−1)=(*n*^2^+2), a condition which can be satisfied only if the dielectric constant becomes infinite. This is now the polarization or dielectric catastrophe, whereby the valence electrons in any constituent atom are now detached from the parent atom and metallization then occurs.

Adopting a model of an isolated atom as a perfectly conducting sphere, it can be shown from electrostatics that its electronic polarizability is *α*=*r*^3^, where *r* is the atomic radius and the molar refractivity is then 
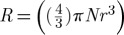
; *R*/*N* can also be taken as the volume of the isolated atoms. On the Goldhammer–Herzfeld view, metallization occurs when the available volume *V* (recall this is the molar volume) becomes equal to, or less than, *R*, the molar refractivity, and the system of localized conducting spheres then becomes one large, macroscopic conductor!

**Figure 8.**
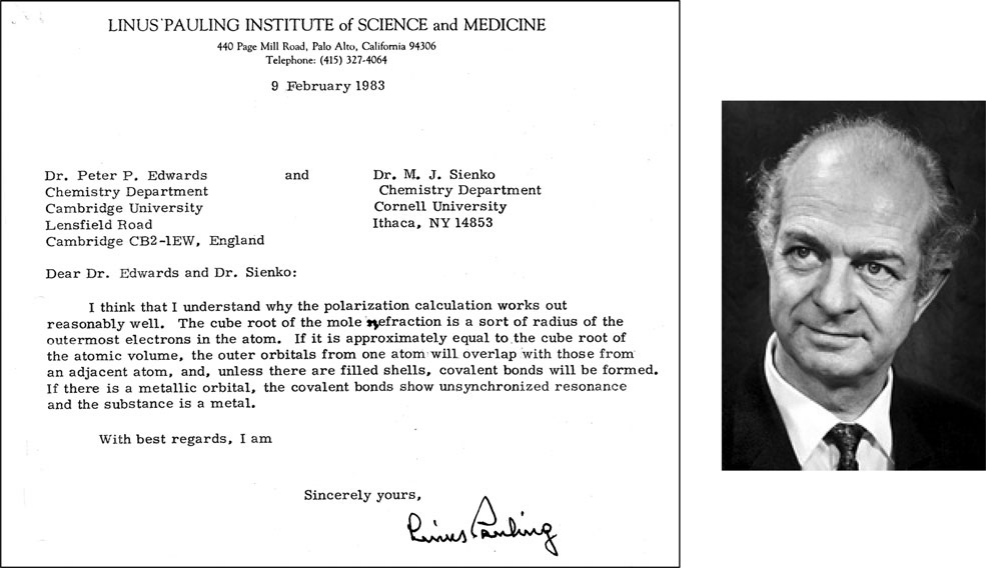
Linus Pauling offers his visionary insights and remarkable intuition into the success of the Goldhammer–Herzfeld criterion in rationalizing the occurrence of metallic character in the periodic table of the chemical elements. He also establishes the direct link with the concept of the metallic orbital (Edwards & Sienko 1983c). Photograph courtesy of California Institute of Technology.

The link from this view of metallization to Pauling’s ‘metallic orbital’ was noted by Linus Pauling himself (Pauling 1983) in a letter to P. P. Edwards and M. J. Sienko on 9 February 1983 (figure 8).

There, Pauling pointed out that an *approximate* measure of the radius of the outermost valence electrons in an isolated atom is the cube root of the molar refractivity (he notes this as ‘the mole refraction…’; figure 8). If this value is approximately equal to the cube root of the molar (‘atomic’) volume in the condensed phase (solid or liquid), then the outer (valence) orbitals from one atom will overlap those from an adjacent atom. Unless these are filled shell orbitals, covalent chemical bonds will be formed. If there is a metallic orbital (partially filled shell orbitals), the covalent bonds will show unsynchronized resonance of electron pair bonds from one interatomic position to another. The resulting resonance stabilization (noted in §2) then gives the system metallic properties.

The link with Mott’s view for the metal–non-metal transition can be seen from the following (Edwards & Sienko 1983*a*). Approached from the non-metallic regime, the transition to the metallic state can be viewed in terms of the Goldhammer–Herzfeld polarization or dielectric catastrophe at *n*_c_, whereby the Coulomb attractive potential *V* (*r*) binding an electron–hole pair now drops to zero, as *V* (*r*)=−*e*^2^/*εr* and as *n*→*n*_c_, 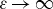. Thus, valence electrons are thereby spontaneously ionized from their parent atoms to form a conduction electron gas and we now have a metallic element. Under such critical conditions, it is the inability of individual atoms to retain their valence electrons in the face of fierce competition from the multitude of other atoms in the condensed phase that leads to a ‘runaway’ increase in electronic polarizability and, ultimately, metallization. Clearly, the larger the (isolated) atom polarizability (*α*), and the greater the elemental density (*n*, the atom number density), the greater the interactions, and more intense the interactions leading to enhanced polarization.

The indicators shown along the atom density axis of figure 7 represent theoretical estimates for the metallization densities of the five different chemical elements derived from the Goldhammer–Herzfeld criterion—recall a prescription dating from 1913 and from 1927 (!), using values of the respective atomic electronic polarizabilities.

One can see that the predicted metallization densities are generally in very good agreement with the experimental elemental densities at which one sees a very rapid increase in the measured electrical conductivities. This agreement is even more pleasing when one realizes that we are witnessing in figure 7 the genesis of the metallic state in elements of the periodic table as chemically and physically diverse as hydrogen, oxygen, rubidium, caesium and mercury.

But how do these critical metallization densities compare with Mott’s *T*=0 K criterion (equation 4.1)? And how do we know that the observed conductivities close to, and above, the ‘Goldhammer–Herzfeld’ line are indeed indicative of the genuine metallic forms of these chemical elements under these extreme conditions?

In the absence of a statement as conclusive and decisive as Mott’s for the case of *T*=0 K (figure 1), how can one precisely define the metallicity of such disordered and diverse elemental systems at very high temperatures? Under these situations, one makes use of an important—and perhaps equally decisive—physical assertion for metallicity first reported by Ioffe & Regel (1960), and developed extensively by Mott (1990). This states that any high-temperature, disordered system will remain metallic—or indeed attain metallic status—if the characteristic mean free path, l, of the valence (conductive) electrons exceeds the mean distance, *d*, between the constituent atoms or molecules providing those carriers of electrical current. The lower limit of the associated electrical conductivity for such a metallic system at *T*=0 K is now recast for the *T*≫0 K case as the Mott–Ioffe–Regel minimum metallic conductivity 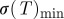, and is taken as the limiting situation in which l≈*d*, the very onset of the metal–non-metal transition (or vice versa). Because of the (obvious) disorder intrinsic within any high-temperature fluid, or vapour, the conduction electrons in this regime—note, still regarded as itinerant (figure 3)—now suffer resistive scattering events at each and every atom or molecule in the fluid. This so-called *strong-scattering* electronic regime, or state, for which l≈*d*, therefore indicates the minimum conductivity which one could anticipate for a disordered, high-temperature metallic fluid under the *T*≫0 K conditions outlined here.

**Figure 9.**
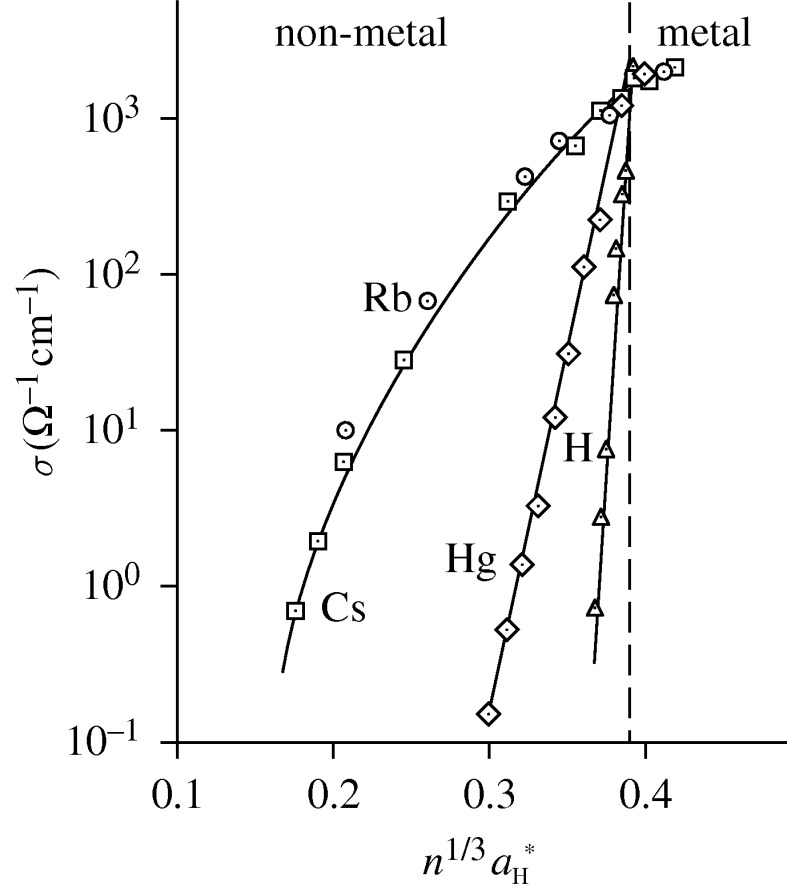
The transition to the metallic state for high-temperature (*T*> 1000 K) fluid caesium, rubidium, mercury and hydrogen: the dependence of the electrical conductivity on the scaling parameter *n*^1/3^*a*_H_^*^. The dotted line drawn at *n*^1/3^*a*_H_^*^ indicates the common metallization condition for these chemical elements. To the left of the metallization condition, we have the non-metallic form of the four elements (non-metallic fluids); to the right, we have the corresponding metallic state (the metallic fluids). Above the metallization threshold, we anticipate conduction based on the theory put forward by Ziman (1961).

This simple but powerful argument leads to an estimate of 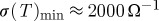 cm^−1^ for the conductivity of fluid hydrogen, rubidium, caesium and mercury at the metallization threshold. Remarkably, also, all of these chemical elements *and* nitrogen and oxygen exhibit essentially the same value of the metallic conductivity at the non-metal to metal transition. In the truly metallic regime, the Ziman theory of conduction in liquid metals appears satisfactory for l>*d* (Ziman 1961). A characteristic atomic property of any chemical element is the radial extent of the electron charge density associated with the outermost (valence) electrons; it is these electrons which ultimately ionize to yield the metallic state. This ‘effective size’ is usually taken as the Bohr-orbit radius of the valence orbit. It is informative to plot the conductivity as a function of *n*^1/3^*a*_H_^*^, where *n* is the atom (valence) electron density (figure 9). In contrast to the metallic regime in which all five of the elements have essentially the same conductivities, the corresponding density dependencies of their electrical conductivities vary quite significantly from each other within the non-metallic regime. There is thus clear scaling behaviour for hydrogen, the alkali metals and mercury.

Thus, metallic conductivities are essentially the same for all these chemical elements, but the density dependence of conductivities within the non-metallic region is determined systematically by the radical extension of their electronic charge distribution of the atoms and their polarizability.

The change in slope at the Mott–Ioffe–Regel value of approximately 2000 Ω^−1^ cm^−1^ indicates that all these elements attain metallic status when 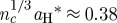, a value not too dissimilar to that derived by Mott in his original discussion of the metal–non-metal transition for hydrogen-like systems. Moreover, Herzfeld (1927) predicted over 80 years ago that compressed hydrogen should undergo a transition to the metallic state at this elemental density. These high-temperature, high-pressure extreme conditions illustrate again that the pivotal role of atomic properties in dictating the status of any chemical element in the periodic table as either a metal or a non-metal.

## 5. The periodic table of the chemical elements

**Figure 10.**
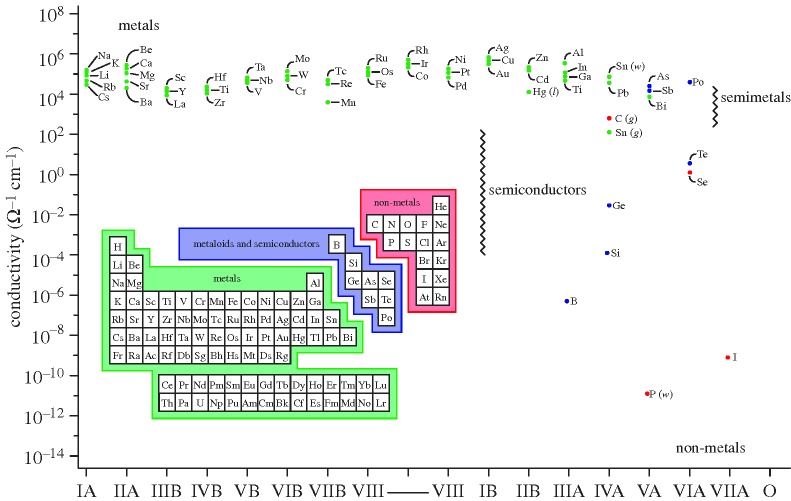
Room-temperature electrical conductivities for the majority of chemical elements of the s-, p- and d-blocks of the periodic table. The inset represents one conventional designation of elements as metals, metalloids and semiconductors, as non-metals. (Adapated from Logan & Edwards 1985.)

It is quite clear that experiments and considerations of the type reviewed here allow us to expand our basic perceptions and definitions of metals and non-metals (Logan & Edwards 1985). Our conventional distinction between these two canonical states of matter within the periodic table of the elements is undoubtedly influenced—one might even go as far as to say programmed—by our experience of the nature of the chemical elements under ambient conditions (generally room temperature, room pressure) on our planet. Without hesitation, one would obviously clarify the elements hydrogen, oxygen and nitrogen as ‘classical’ non-metals and the elements rubidium, caesium and mercury as ‘prototypical’ metals. Thus, armed even with room temperature conductivity (resistivity) data (figure 10), one can fairly easily identify elements of the periodic table for which the appellation ‘metal or non-metal?’ is most appropriate (as noted earlier, these differences in conductivities are even more exaggerated at low temperatures). Crystal structures for elements across the periodic table also readily give a demarcation into metals, metalloids and semiconductors and non-metals.

**Figure 11.**
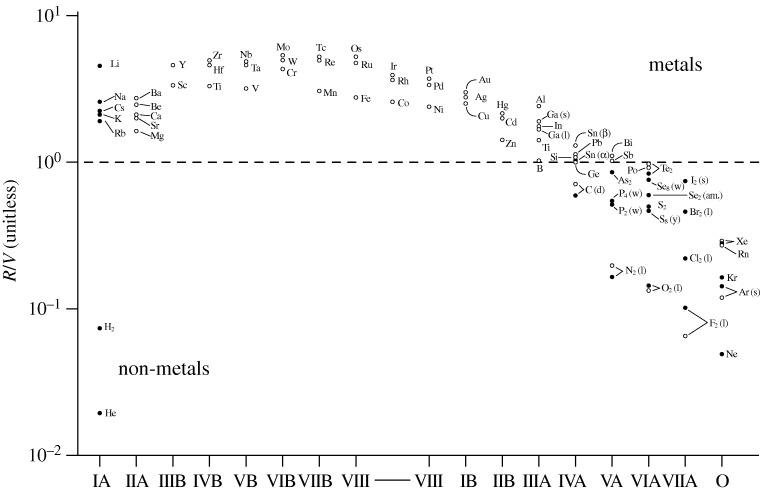
Metallization of elements of the periodic table under ambient conditions imposed on the Earth. The figure shows the ratio (*R*/*V*) for elements of the s-, p- and d-blocks of the periodic table. Here, *R* is the molar refractivity and *V* is the molar volume. The shaded circles represent elements for which both *R* and *V* are known experimentally. The open circles are for elements in which *R* is calculated and *V* is known experimentally. (Adapted from Edwards & Sienko 1982, 1983*b*).

Using the Goldhammer–Herzfeld criterion with measured atomic electronic polarizabilities and condensed phase molar volumes allows one to readily predict which elements are metallic, which are non-metallic, and which are borderline when in their condensed phases (solid or liquid). Just how well the overall features of the periodic table conform to the simple criterion (*R*/*V*) can be seen by referring to figure 11, which shows the data for the s-, p- and d-block elements at their normal densities (Edwards & Sienko 1982, 1983*b*).

**Figure 12.**
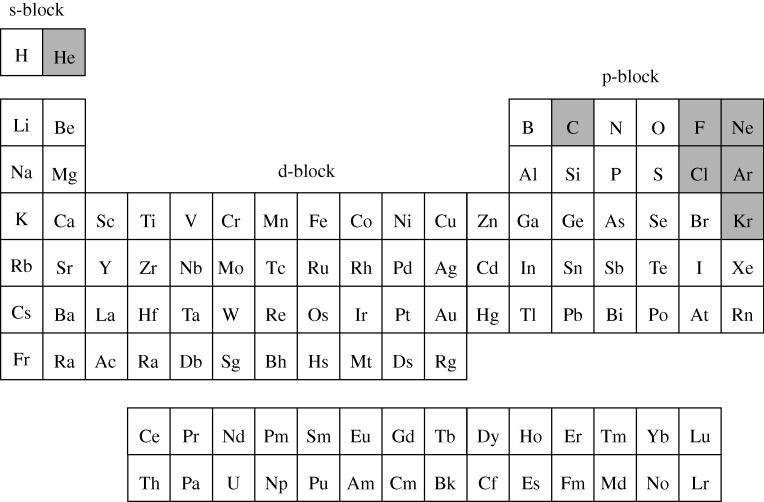
A possible form of the periodic table of the chemical elements at a pressure close to 3 million atmospheres. This table is not yet complete—certain elements have not been investigated to these high pressures (e.g. F_2_, etc.). Notice now the absence of the traditional demarcation separating metals from non-metals (grey background) under normal conditions on Earth. Fluid hydrogen is shown as a metal; solid hydrogen does not attain metallic states, even at these high-imposed pressures. A new development could be that certain prototypical ‘metals’ (e.g. Li) could become non-metals at these high pressures (Ashcroft 2009)! We do not discuss such a possibility here. (Adapted from Edwards & Hensel 2002.)

The continued success of the criterion, even in its simplest form, illustrates the veracity of the Goldhammer–Herzfeld view of the over-riding importance of *atomic* properties in dictating the very nature—metal or non-metal—in the condensed state; thus the instantly recognized ‘diagonal’ demarcation separating metals from non-metals in the periodic table is taken as the cornerstone of our conventional classification of the chemical elements (figure 12).

The relatively small value of the polarizability of atomic hydrogen (0.67 Å^3^) then indicates that very high elemental densities are required for the (pressure induced) transition to the metallic state of hydrogen. In contrast, the high polarizabilities of rubidium (47.3 Å^3^) and caesium (59.7 Å^3^) ensure that these elements are naturally metallic at ambient conditions on the surface of the Earth. As Herzfeld pointed out in 1927, if mercury had, in the solid or liquid state, the large molar volume of, say, an alkali atom (e.g. potassium, approximately 45 cm^−3^), then it would not be a metal (Herzfeld 1927). The striking manifestation of the non-metallic state of ‘expanded’ mercury is testament to this critical aspect (figure 7).

The classification ‘metal or non-metal?’ is therefore not an inherent and unchanging property for any element, or indeed any substance, material or system. For the chemical elements of the periodic table, density is indeed a critical thermodynamic parameter governing either their metallic or their non-metallic status; the data reviewed here surely represent a remarkable manifestation of that fact.

The transition from non-metallic to metallic hydrogen at high pressures of several megabars also has far-reaching consequences for the interior of giant planets such as Jupiter, Saturn or Jupiter-like extrasolar planets that have been detected in great number since 1996. Hydrogen and helium are by far the most abundant elements in nature and contribute about 98 per cent to their planetary masses. The transition from an outer fluid envelope with cool molecular, non-conducting hydrogen to an inner fluid envelope composed of warm atomic, conducting hydrogen occurs at 1–2 Mbar inside Jupiter, i.e. between 80 and 90 per cent of its radius (Nellis *et al.* 1996). This non-metal to metal transition has long been discussed as a candidate to explain the existence of a layer boundary if it is accompanied by a simultaneous thermodynamic instability, the plasma phase transition (Stevenson 1982; Guillot 1999). This problem was raised first by Landau & Zeldovich (1943) and is still under lively discussion (Redmer *et al.* 2010).

Today, *ab initio* molecular dynamics simulations are performed routinely to study warm dense matter, the electronic properties of warm dense hydrogen (Holst *et al.* 2008) and hydrogen–helium mixtures (Lorenzen *et al.* 2009) in particular. This approach treats the electron correlations within finite-temperature density functional theory and considers the disordered liquid structure based on the Born–Oppenheimer approximation. As a result, the non-metal to metal transition in warm dense hydrogen as derived from shock wave experimental data on the electrical conductivity (Weir *et al.* 1996) and reflectivity (Celliers *et al.* 2000) is well reproduced at the condition of about *n*^1/3^_c_*a*_H_∼0.38.

**Figure 13.**
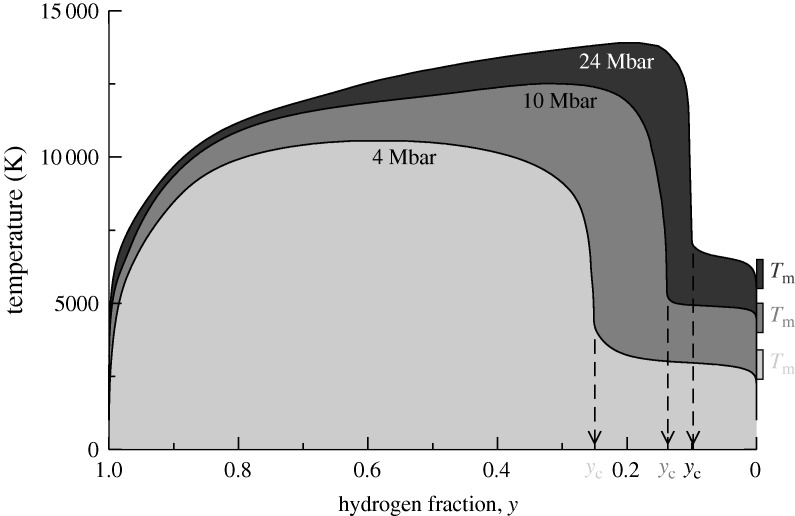
Miscibility gap in the hydrogen–helium system for constant pressures as a function of the hydrogen concentration, *y*=*N*_H_/(*N*_H_+*N*_He_) (see Lorenzen *et al.* 2009). The calculated melting temperatures, *T*_m_, of solid helium are indicated on the right-hand side for each pressure. The strong increase in the demixing temperatures occurs at critical hydrogen concentrations *y*_c_, which are in accordance with Mott’s criterion, 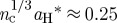. We conclude that the thermodynamics that drives the phase separation in the mixture is caused by a continuous non-metal to metal transition in the hydrogen subsystem.

Even more challenging, hydrogen–helium mixtures at high pressures were studied with *ab initio* molecular dynamics simulations in order to reveal the existence and location of a possible demixing region which is important for the luminosity and, thus, for the evolution and cooling behaviour (age) of Jupiter-like giant planets. It is astonishing that Mott’s simple criterion of a minimum metallic conductivity also applies to such complex mixtures at high temperatures of about 1 eV (the temperatures in the expanded metals discussed above are only about 0.2 eV). The *ab initio* equation of state data displayed in figure 13 and electrical conductivities derived from a Kubo–Greenwood formula indicate that helium demixes from hydrogen above 1 Mbar at a critical hydrogen concentration of *n*^1/3^_c_*a*_H_∼0.25—which is just the original Mott criterion for metallization according to equation (4.2) (Lorenzen *et al.* 2009)! These new results have the potential to unravel the mystery of Saturn’s excess luminosity, which can be explained by the formation of helium droplets and their descent in almost the entire interior of that planet above 1 Mbar—which is clearly a result of metallization in the hydrogen subsystem. We conclude that the ideas of Sir Nevil Mott are of paramount importance to understand the behaviour of matter not only at *T*=0 K on Earth but also at substantial thermal energies and pressures inside giant planets in the solar system and beyond.

It now seems quite clear, moreover, that within the interiors of the giant planets, Jupiter, Saturn, etc., our periodic table would appear quite different; one possible form at 3 million atmospheres is shown in figure 12. Here, we see no evidence of the (Earthly) instantly recognizable, diagonal demarcation line within the p-block separating metals from non-metals, and the vast majority of all the chemical elements are now metals. Perhaps, we should recast Sir Nevill Mott’s enquiry (figure 1) ‘What is a metal?’ rather to that of … ‘When is a metal?’!

## 6. Epilogue

In 1949, Sir Nevill proposed a model, as Ashcroft (1993) has noted, ‘deceptive in its simplicity’, which demonstrated the essential, critical importance of both electron–electron correlation and screening effects in the basic physics underlying the transition of a system from the non-metallic to the metallic state. Approached from the metallic regime, all carriers are trapped to yield the non-metallic, non-conducting state at *T*=0 K.

Approached from the non-metallic state, arguments by Goldhammer (1913) and Herzfeld (1927), which are even older than Mott’s, lead to the picture of the wholesale freeing of all localized carriers, signifying the onset of a metallic state, and Pauling’s equivalent metallic orbital.

We hope to have illustrated that these contributions are typical of Sir Nevill Mott’s approach to research—a determination to get to the underlying physics and place this within tractable models, for both theorist *and* experimentalist—and his antipathy for unnecessary formalisms.

Although the precise details of the progression of metal to non-metal may surely involve physics significantly more complex—particularly at the very transition itself—both the Mott and Goldhammer/Herzfeld views signifying the onset of the metallic/non-metallic states must be essentially correct. In addition, these descriptions provide powerful indications not only to the location and characteristics of the transition itself, but also to remarkable insights into the underlying reasons why ‘… a metal conducts and a non-metal doesn’t’.

## Footnotes

1 Following Mott, we designate metals and non-metals as the states of matter at *T*=0 K (excluding superconductors). Thus, at absolute zero, insulators and semiconductors are non-metals.

2 This relates to a discussion of the paper cited in de Boer & Verwey (1937).
